# Effect of Si(111) Surface Modification by Ga Focused Ion Beam at 30 kV on GaAs Nanowire Growth

**DOI:** 10.3390/ijms24010224

**Published:** 2022-12-23

**Authors:** Nikita Shandyba, Sergey Balakirev, Vladislav Sharov, Natalia Chernenko, Danil Kirichenko, Maxim Solodovnik

**Affiliations:** 1 Institute of Nanotechnologies, Electronics and Equipment Engineering, Southern Federal University, Taganrog 347922, Russia; 2 Laboratory of Renewable Energy Sources, Alferov University, Saint Petersburg 194021, Russia; 3 Laboratory of Surface Optics, Ioffe Institute, Saint Petersburg 194021, Russia

**Keywords:** molecular beam epitaxy, A3B5, nanowires, focused ion beam, surface modification, nanostructure, self-catalytic growth

## Abstract

This paper presents the results of experimental studies of the effect of Si(111) surface modification by Ga-focused ion beam (FIB) at 30 kV accelerating voltage on the features of the epitaxial GaAs nanowire (NW) growth processes. We experimentally established the regularities of the Ga ions’ dose effect during surface modification on the structural characteristics of GaAs NW arrays. Depending on the Ga ion dose value, there is one of three modes on the surface for subsequent GaAs NW growth. At low doses, the NW growth is almost completely suppressed. The growth mode of high-density (up to 6.56 µm^−2^) GaAs NW arrays with a maximum fraction (up to 70%) of nanowires normally oriented to the substrate is realized in the medium ion doses range. A continuous polycrystalline base with a dense array of misoriented short (up to 0.9 µm) and thin (up to 27 nm) GaAs NWs is formed at high doses. We assume that the key role is played by the interaction of the implanted Ga ions with the surface at various process stages and its influence on the surface structure in the modification region and on GaAs NW growth conditions.

## 1. Introduction

The great interest to nanowires (NW) based on A3B5 semiconductors is caused not only by the combination of their unique optical and electrical characteristics due to their structural features, but also by their possibility of direct integration with silicon. This approach makes it possible to create functional materials based on A3B5 semiconductors with high structural perfection on a cheap silicon substrate. This, in turn, opens wide opportunities in the field of development and creation of advanced microelectronics and nanophotonics systems, which combine the advantages of both platforms—silicon and A3B5. To date, A3B5 NWs on silicon substrates have already proven themselves in some areas, such as creation of high-efficiency transistors [1], laser structures with a low threshold power [2], photodetectors [3], solar cells [4], etc.

The creation of NW-based devices requires the development of methods for precision control and management of key characteristics (length, diameter, density, etc.), including their location on the surface. At the same time, methods based on the vapor–liquid–solid mechanism dominate among epitaxial approaches for obtaining NWs. Considering this fact, the control of such structures’ characteristics is largely related to the control of the catalytic centers’ (catalyst droplets) ensemble parameters, regardless of whether there is self- or hetero-catalytic growth [5,6]. Therefore, the main approach that makes it possible to control the NW arrays’ parameters, including their location, lies in preliminary surface structuring in order to localize nucleation centers (catalyst droplets), as a rule, in the windows of the masking layer [7] using lithographic methods. The most widespread among them are electron-beam lithography [8,9], nanoimprint lithography [10,11], and nanospheric lithography [12,13]. However, these methods have several disadvantages [14], including poor compatibility of liquid and/or plasma processing operations with subsequent epitaxial growth [15,16].

A promising alternative to traditional lithographic methods is the technique of surface pre-growth treatment based on local ion-beam modification of the substrate using a focused ion beam [17,18,19,20,21,22,23,24]. The main advantages of this method include the possibility of carrying out technological operations of local ion-beam etching with high spatial resolution under high vacuum conditions, without the need to use resists, masks, and chemical etching [25]. This method can be used to form A3B5 NWs in two ways. The first one is based on the formation of nanosized windows in the masking oxide layer in a strictly defined region, followed by the localization of catalyst droplets in them and the NW growth [18,20]. The second one is based on local implantation of ions into the substrate surface with subsequent annealing, which leads to the formation of metal catalyst droplets at the sites of beam impact due to the segregation of implanted material atoms [17,19,21,22,23,24]. By varying technological parameters of the FIB treatment and pre-growth annealing, it is possible to effectively control the size, density, and position of the formed metal droplets, thereby largely predetermining the characteristics of subsequently growing NWs. However, despite the large number of works on this topic, many questions related to the mutual influence of the ion-beam processing main parameters (dose, accelerating voltage, beam current, processing topology, etc.) and epitaxial growth (temperature and time of annealing and growth, the ratio of growth components, etc.) on the key NW characteristics are still poorly understood.

In this work, we show that the effect of the ion dose during surface FIB treatment on the processes of the subsequent NW growth cannot be explained only by the segregation effect of embedded Ga ions with the catalyst droplets’ formation. The results of experimental studies demonstrate that the epitaxial growth of NWs within modified Si(111) regions can proceed according to three scenarios with significantly different results, depending on the dose of the implanted Ga ions. In this case, the key influence on the NW growth processes is determined by the interaction of implanted Ga ions with the substrate surface at the stages of FIB modification and pre-growth UHV annealing and their effect on the structure of the substrate surface in the modification region.

## 2. Results and Discussion

### 2.1. GaAs Nanowire Growth within FIB-Modified Areas

**Figure 1:**
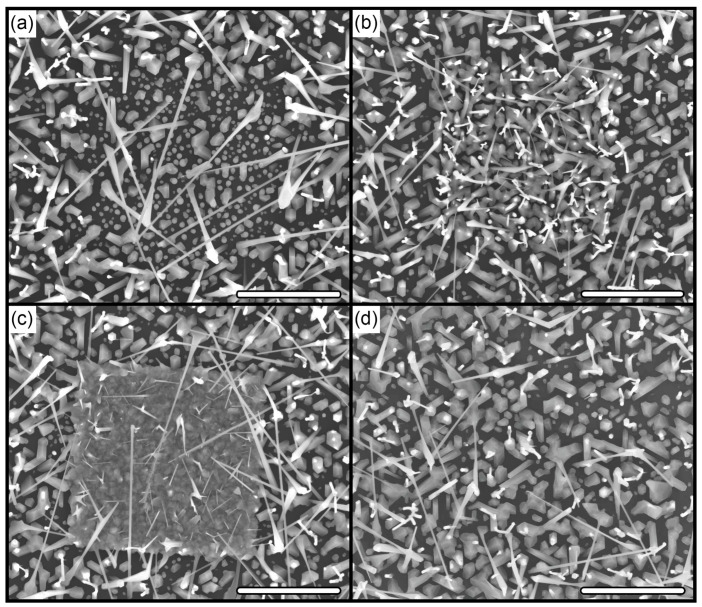
SEM images (view from above) of modified areas with Ga ion dose of 0.052 (**a**), 0.26 (**b**), 10.4 (**c**) pC/µm^2^, and unmodified area (**d**) after GaAs NW growth. Scale bar is 3 µm.

**Figure 2:**
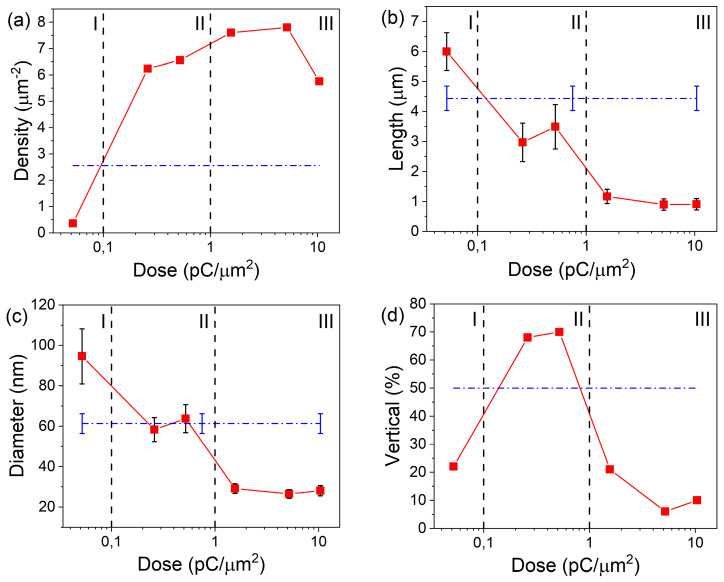
Dependences of the density (**a**), length (**b**), diameter (**c**), and vertical orientation of NWs (**d**) on Ga ion dose (dash lines correspond to values for unmodified areas).

Analysis of SEM images of the obtained samples showed a significant effect of the Si(111) substrates surface FIB modification on the formation of GaAs NW and, as a result, their geometrical characteristics (Figure 1). The dependences of the GaAs NW array main parameters (density, average length and diameter, and the proportion of vertical (normally oriented to the substrate) NWs) on the Ga ion dose during FIB processing are shown in Figure 2. The obtained data are based on a quantitative analysis of the structure geometrical characteristics according to the SEM images. As can be seen in Figure 1, they have a rather complex, non-linear character. Comparison of the SEM images and the obtained dependences allow us to distinguish three conditional areas corresponding to different ion dose range under consideration (Figure 2)—area I, area II, and area III. Each of them is characterized by its own type of the structure morphology (Figure 1a–c, respectively) and a set of geometrical parameters. In this case, we can talk about the implementation of its own set of growth conditions in each of these areas, i.e., modes of GaAs NW growth.

***Area I—low ion dose values (from below 0.052 pC/µm^2^ to ~0.1 pC/µm^2^).*** There is an almost complete suppression of the NW growth and a significant suppression of parasitic nanocrystal formation in this region (Figure 1a). The density of GaAs NWs in this area is about 0.36 µm^−2^, which is almost an order of magnitude (~7 times) lower than the values for the unmodified surface (2.56 µm^−2^) (Figure 2a). This is a very unexpected result because after 48 min of the growth under arsenic-enriched conditions (see Section 3, Materials and Methods), a high-density (up to~13.6 µm^−2^) array of Ga droplets with an average size of 91 ± 13 nm is observed (Figure 1a). That is, even though that catalyst droplets have formed in this region, NWs do not generally grow from them. In this case, as follows from the data in Figure 2b,c, the average values of the NW diameter and length (95 ± 14 nm and 6 ± 0.6 µm, respectively) in this region exceed the values for the NW array on the unmodified surface (61 ± 5 nm and 4.44 ± 0.4 µm). It is also worth noting the misorientation of NWs: the proportion of normally oriented NWs is about 20%, which is significantly lower than 50% for an unmodified surface.

***Area II—medium ion dose values (from ~0.1 pC/µm^2^ to ~1.0 pC/µm^2^).*** An increase in the dose of ion-beam treatment above ~ 0.1 pC/µm^2^ leads to a significant (by a factor of ~2.5) rise in the density of GaAs NWs within the modification region (up to 6.24–6.56 µm^−2^, see Figure 2a) compared to the unmodified surface (2.56 µm^−2^). In this case, the catalyst droplets on the substrate surface disappear, but the parasitic growth of GaAs nanocrystals in the base and between NWs is activated (Figure 1b). It is important to note that the NW diameter (58 ± 6–64 ± 7 nm) and length (2.97 ± 0.64–3.49 ± 0.74 µm) remain almost the same as on the unmodified surface in this dose range (Figure 2b,c), even with a multiple (by a factor of ~2.5) increase in the NW density. In this case, the proportion of NWs normally oriented to the substrate increases up to 70% (50% for the unmodified surface).

***Area III—high ion dose values (from ~1.0 pC/µm^2^ and above).*** This range of FIB doses is characterized by the formation of a continuous (100% filling) polycrystalline base with an array of misoriented thin GaAs NWs (Figure 1c), which may indicate a radical change in the local growth mechanism within these areas. The density of GaAs NWs continues to increase, reaching a maximum value of 7.8 µm^−2^ (at 5.21 pC/µm^2^), and then begins to decrease (5.76 µm^−2^ at 10.4 pC/µm^2^). In contrast to the previous case, an increase in the density is accompanied by a decrease in the NW length and diameter with stabilization of values in the ranges of 0.9–1.17 µm and 27–29 nm, respectively, starting from a dose of 1.56 pC/µm^2^ (Figure 2b,c). The proportion of vertical NWs within this region is also stabilizing at the level of 6–10%.

An analysis of the GaAs NW length and diameter distribution shows that an increase in the FIB treatment dose leads to a shift in the distribution ranges from large values to smaller ones (Figure S1). In addition, in area I and area III, a significant narrowing of the ranges of measured values is observed not only in comparison with area II, but also in comparison with the unmodified surface. Even though the data from area I are not enough to estimate the true distribution (too small sample volume), in general, it can be generally said that the FIB surface modification makes it possible to modulate the shape of the NW characteristics distribution in a quite wide range of values.

As can be seen from the presented data, such an unusual and complex character of the FIB treatment dose effect on the nanostructures growth processes and their characteristics cannot be explained by the effect of the embedded Ga atoms’ (ions) segregation alone, which leads to the formation of catalyst droplets on the modified surface. Obviously a more detailed analysis of the specifics of the Ga ions’ interaction with the surface and Si substrate crystal structure is required at different levels of FIB exposure intensity, as well as at different processing stages: modification, high vacuum annealing, and epitaxial growth.

### 2.2. FIB Modification Effect on Si(111) Surface

**Figure 3:**
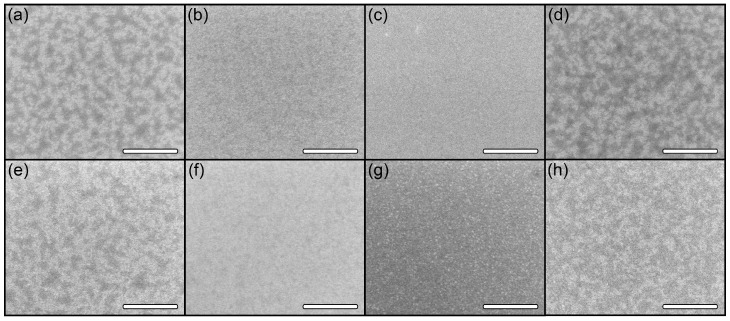
SEM images of modified areas before (**a**–**d**) and after (**e**–**h**) annealing stage for Ga ion doses (the dose is expressed in pC/µm^2^) of 0.052 (**a**,**e**), 0.26 (**b**,**f**), and 10.4 (**c**,**g**) and the unmodified area (**d**,**h**). Scale bar is 300 nm.

Analysis of SEM images of the surface immediately after FIB treatment (Figure 3a–c) did not reveal any significant morphological features, which is associated with the choice of FIB treatment modes that minimize etching processes. For comparison, Figure 3d shows an image of the Si(111) surface without modification. However, analysis of the image contrast (based on the secondary electron signal) shows that as the treatment dose is increased, the contrast within the modification area levels out becoming more uniform compared to the unmodified surface (Figure 3a–c). The similarity of the image contrast in Figure 3a,d may indicate that the FIB treatment in the low-dose region (area I) does not seem to have any significant effect directly on the surface. We assume that the structure of native oxide on the Si surface remains almost unchanged. As the dose is increased (Figure 3b,c—area II and area III), the contrast within the area of modification becomes more and more uniform. This may be due to both active modification (material mixing) of the near-surface layer and the accumulation of Ga ions near the surface, which generates an additional signal of secondary electrons that align the contrast. In any case, Ga droplets or any other inclusions are not detected on the surface even at the maximum treatment doses.

The surface morphology of the same FIB-modified regions after annealing is shown in Figure 3e–h. As can be seen in the presented data, in area I (Figure 3e) and area II (Figure 3f), the contrast structure underwent no significant changes, with the only exception that the proportion of light tones increased. This may indicate additional surface charging, which is characteristic of areas with reduced conductivity. At the same time, in area III (Figure 3g), a high-density array of nanosized Ga droplets appeared within the modified region, which is due to the segregation of embedded Ga ions during annealing.

**Figure 4:**
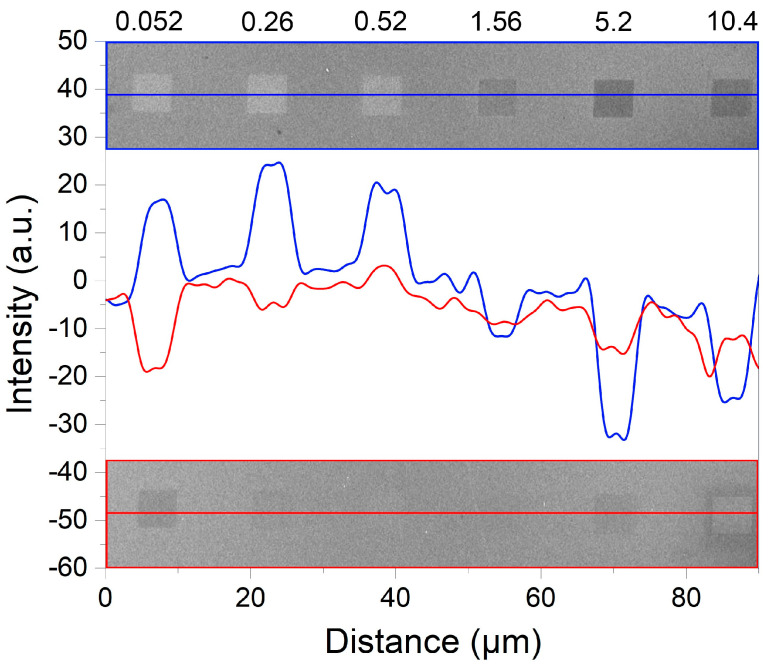
Dependence of the SEM-contrast intensity for each modification area before (**bottom**) and after (**top**) annealing. The top axis shows the doses of Ga FIB treatment (in pC/µm^2^).

Figure 4 shows the dependences of contrast intensity for FIB-modified regions before and after annealing obtained from the signal of secondary electrons. It is clearly seen how the tone of the contrast is inverted from dark (negative intensity values) to light (positive intensity values) after annealing in the range of low and medium doses (up to ~1.0 pC/µm^2^—area I + area II). In this case, area I (corresponding to region 0.052 pC/µm^2^ in Figure 4) stands out by the maximum difference in intensities before and after annealing. At the same time, the opposite picture is observed in the region of high doses (area III, from 1.0 pC/µm^2^)—a significant increase in the intensity of the dark tones.

It is well known that the intensity of the secondary electrons signal depends on many factors, including the surface conductivity. In this case, a darker tone of the image corresponds to higher conductivity of analyzed area in the absence of other contributions [26]. Thus, it can be assumed that during annealing in the range of low (area I) and medium (area II) doses, the near-surface layer becomes less conductive in comparison not only with the surrounding regions of the unmodified surface, but also with the same regions immediately before annealing. This may indicate both the complete segregation of the embedded Ga ions and the restructuring of native Si oxide on the surface during its interaction with Ga atoms diffusing from the near-surface layer [24]. At the same time, a significant amount of Ga ions remains in the near-surface layer in area III, due to considerably (by orders of magnitude) high treatment doses. This aspect modulates the local conductivity of the substrate material, and the oxide layer on the surface is either absent or its thickness is negligibly small.

**Figure 5:**
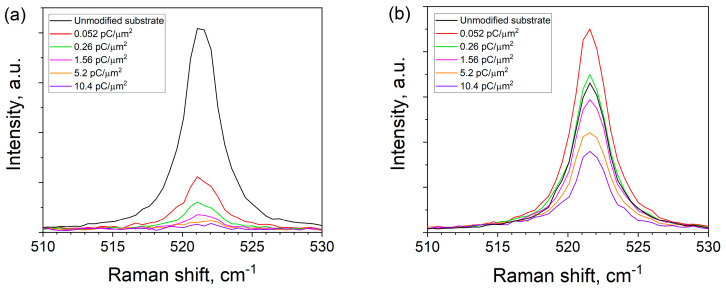
Raman spectra of substrate areas with different ion dose before (**a**) and after (**b**) UHV annealing.

These assumptions correlate with the results of Raman spectroscopy. As can be seen from the data in Figure 5a, an increase in the dose during the FIB modification, as expected, leads to both a general decrease in the intensity of the spectrum and a decrease in the intensity of the TO-phonon line (~521 cm^−1^). This is due to the disordering of the crystal structure in the region of the beam impact, the degree of which is proportional to the total dose of implanted Ga ions. After annealing, the intensity of the Si TO mode at low and medium doses (area I and II) is comparable to the values of the unmodified surface or even exceeds them. At the same time, in the region of high treatment doses, the intensity remains significantly below the reference values (Figure 5b), which indicates an incomplete restoration of the crystal structure after FIB modification. This means that a significant number of Ga atoms remains in the near-surface layer, which did not have enough time to leave the Si lattice during annealing. This correlates well with both surface morphology data (Figure 3g) and image contrast analysis results (Figure 4).

### 2.3. Proposed Mechanisms of GaAs NW Growth within FIB-Modified Areas

The analysis and systematization of the obtained data suggest three scenarios for the development of the system under consideration, depending on the ion-beam treatment dose.

#### 2.3.1. Area I (Ion Dose Is from Below 0.052 pC/µm^2^ to ~0.1 pC/µm^2^)

In the low-dose region, the implantation of Ga ions predominantly occurs, without significant interaction with the surface oxide (considering an energy of 30 keV). This is supported by the similarity of the SEM images in Figure 3a (with modification) and 3d (without it). The ions’ implantation, on the one hand, leads to the generation of defects (the TO peak in Figure 5a decreases) and, on the other hand, it modulates the conductivity of the near-surface layer, which leads to a darkening of the region in SEM images in secondary electrons at low doses (Figure 4). At the annealing stage, the embedded Ga atoms diffuse from the volume to the surface, where they can either desorb or interact with the native Si oxide film. In the second case, this interaction can form a Ga-O-Si complex oxide film, which can have a masking effect. The interaction of Ga atoms with SiO_x_ is supported by an increase in the intensity of light tones of the modified region in SEM images in secondary electrons (Figure 4). In any case, the crystalline structure of the modified area is restored (Figure 5) and, subsequently, does not have any effect on the NW growth. It can be assumed that the oxide film modified with Ga atoms on the substrate surface becomes more chemically and thermally stable due to enrichment in the Ga_x_O_y_ component [27]. Then the formed film enhances the masking effect of the oxide suppressing (leading to a breakdown) the processes of epitaxial growth due to the desorption of growth components from the oxide surface and/or their migration to unmodified regions where the nucleation threshold is lower due to the pore formation in SiO_x_ at the annealing stage [28]. This assumption explains the almost complete suppression of the growth of both GaAs NWs and parasitic nanocrystals (see Section 2.1). The formation of the Ga nanodroplet array (Figure 1a) apparently already occurs in the growth process due to a significant difference in the sticking coefficients and lifetime in the adsorbed state of Ga atoms and As molecules at 600 °C on the oxide surface. In any case, this issue requires separate additional research.

#### 2.3.2. Area II (Ion Dose Is from ~0.1 pC/µm^2^ to ~1.0 pC/µm^2^)

In the range of medium FIB treatment doses, the situation is generally similar to that described above. However, at the FIB processing stage, the interaction of beam ions with the surface is enhanced, which leads to a more uniform image contrast of the site surface in secondary electrons (Figure 3b). In this case, even though the defectiveness of the substrate in the modification area is significantly higher (Figure 3a), the crystal structure is also completely restored during annealing (Figure 3b). Since the number of Ga atoms diffusing to the surface is much larger, two processes occur in the system. On the one hand, the native Si oxide film is modified according to the mechanism proposed above (Figure 4). On the other hand, excess Ga forms a network of pores in the oxide layer like that in a native oxide film due to the etching mechanism [29,30]. The presence of pores in a film (relatively resistant to external influences) stimulates the formation of an array of nanosized Ga droplets at the initial stage of growth, from which NWs then develop. As a result, a high-density array of normally oriented GaAs NWs is formed (Figure 1b and Figure 2).

#### 2.3.3. Area III (Ion Dose Is from ~1.0 pC/µm^2^ and Above)

A further increase in the FIB treatment dose leads to the formation of a high-density array of nanosized (10–15 nm) Ga droplets due to the segregation effects at the annealing stage (Figure 3g). At the same time, the modified region does not have time (1 h annealing stage) to completely restore during the annealing process. Thus, by the beginning of epitaxial growth, it contains enough embedded Ga atoms, which is confirmed by contrast data (Figure 4) and Raman spectroscopy (Figure 5a). The presence of a dense array of catalytic centers leads to a complete change in the growth mechanism. During the pre-growth exposure of the substrate to the As flux, droplets crystallize and an array of GaAs nanocrystals is formed, which is a parasitic phase from the point of view of NW growth. Several competing processes take place simultaneously after the growth start. On the one hand, the size of GaAs nanocrystals formed at the exposure stage increases (proliferate) consuming most of the epitaxial material. On the other hand, there is a flow of excess Ga atoms due to the continuing effects of segregation and the presence of oxide residues on the surface between the crystallites. This additional flow can lead to the secondary formation of catalyst centers and the subsequent start of NW growth. This leads to the fact that the NWs diameter and length cease to depend on the FIB treatment dose. This may be because the NWs nucleation and growth on surfaces with complex morphology are primarily determined by the number of sites favorable for these processes, and an increase in the implantation dose leads to an increase in the continuity of the polycrystalline base and a decrease in the number of such sites. Since the additional flow of Ga atoms is rather small, the NWs also have a smaller diameter due to the small size of the catalyst droplets (Figure 2). The competition between the growth processes of the 3D phase (GaAs nanocrystals) and NWs leads the fact that the length of NWs also sharply decreases in comparison with the unmodified surface. The random orientation of NWs relative to the substrate (Figure 2 and Figure 1d) may be due to the formation of catalyst droplets either between GaAs crystallites or on their faces.

## 3. Materials and Methods

### 3.1. Sample Preparation

Epi-ready Si(111) p-type wafers were used as the substrate. At the first stage, ion-beam surface treatment was performed at various FIB parameters to form the required areas. Then, the modified samples were annealed, and GaAs NWs were grown using the molecular beam epitaxy (MBE) technology. The control of the obtained structures morphology was carried out by SEM methods.

Ion-beam treatment of the Si(111) surface was carried out using a Nova NanoLab 600 electron microscope equipped with a FIB system with a Ga ion source. The FIB modification regions were 5 × 5 µm squares. The surface was modified at an accelerating voltage of 30 kV and an ion-beam current of 30 pA. The dose was varied by changing the passes number of the ion beam according to the topology specified by the template. The dose of Ga ions varied from 0.052 pC/μm^2^ to 10.4 pC/μm^2^.

Annealing and self-catalytic GaAs NW growth were carried out on a SemiTEq STE 35 MBE system. Si(111) samples with FIB-modified surface areas were first annealed under ultra-high vacuum conditions at a temperature of 600 °C for 60 min in an MBE growth chamber. Prepared samples were analyzed by SEM and Raman spectroscopy and then used for the subsequent NW growth. The growth of GaAs NWs was carried out at the annealing temperature by deposition of GaAs at an equivalent growth rate of 0.25 ML/s for 48 min with preliminary exposure to an As flux. The Ga and As fluxes were preliminarily calibrated on the GaAs growth rate on GaAs(001) substrates.

### 3.2. Sample Characterization

Quantitative analysis of the NW main parameters was based on SEM images. In this case, to determine the values of the NW length and diameter and to calculate the density and verticality (the proportion of normally oriented NWs from their total number), images obtained at an angle of 38° and 90° (with respect to the substrate plane), respectively, were used. All measurements were carried out using specialized software SIS Software Scandium. The diameter was determined mainly near NW tops.

The intensity distributions of the SEM contrast were plotted on the basis of the analysis of the secondary electron signal from each modified region in the SEM images obtained at an accelerating voltage of 10 kV and an electron beam current of 0.54 nA.

The Raman spectra were measured at room temperature using a Horiba LabRam HR800 setup equipped with a 532 nm diode-pumped YAG laser, a spectrograph with a focal length of 800 mm, and an 1800 mm^−1^ diffraction grating with a spectral resolution of 0.5 cm^−1^. The measurements were carried out in a back-scattering geometry. The beam was focused into a spot about 1 μm in diameter using a ×50 lens with aberration correction. A high-precision piezoelectric scanner was used to position the exciting spot in the region of sample surface modification.

## 4. Conclusions

Thus, we have shown that, by varying the FIB treatment dose of the Si(111) surface, we can control various parameters of GaAs NWs (length, diameter, density, orientation) in a wide range of values. The complex character of the NW main parameters’ dependence on the parameters of the FIB treatment was found and determined from the complete suppression of the NW growth to the formation of polycrystalline structures with misoriented NWs. It is shown that this is due to a change in the modes and mechanisms of the catalytic center formation and the initial stage of NW growth. In this case, the regularities found cannot be explained only by the effects of embedded Ga atoms’ segregation. Based on the obtained experimental data, it was concluded that the NW growth is significantly influenced by the features of the embedded Ga atoms’ interaction with native Si oxide on the substrate surface in the modification region. The possibility of NW arrays’ local formation with different characteristics in a single growth process was experimentally shown.

## Data Availability

Not applicable.

## Supplementary Materials

The following supporting information can be downloaded at: https://www.mdpi.com/article/10.3390/ijms24010224/s1.
