# Supplementary material for: Effect of Si(111) Surface Modification by Ga Focused Ion Beam at 30 kV on GaAs Nanowire Growth
Source: Int J Mol Sci. 2022 Dec 23;24(1):224. doi: 10.3390/ijms24010224 (PMC9820241; doi:10.3390/ijms24010224)
Supplement: Supplementary file 1 [file ijms-24-00224-s001.zip › ijms-2075970-supplementary.pdf]

Effect of Si(111) surface modification by Ga focused ion beam at 30 kV on GaAs nanowire growth

Nikita Shandyba [1], Sergey Balakirev [1], Vladislav Sharov [2], Natalia Chernenko [1], Danil Kirichenko [1] and Maxim Solodovnik [1,*]

[1] Institute of Nanotechnologies, Electronics and Equipment Engineering, Southern Federal University, Taganrog 347922, Russia;

[2] Alferov University, Saint Petersburg 194021, Russia

[3] Ioffe Institute, Saint Petersburg 194021, Russia

* Correspondence: solodovnikms@sfedu.ru

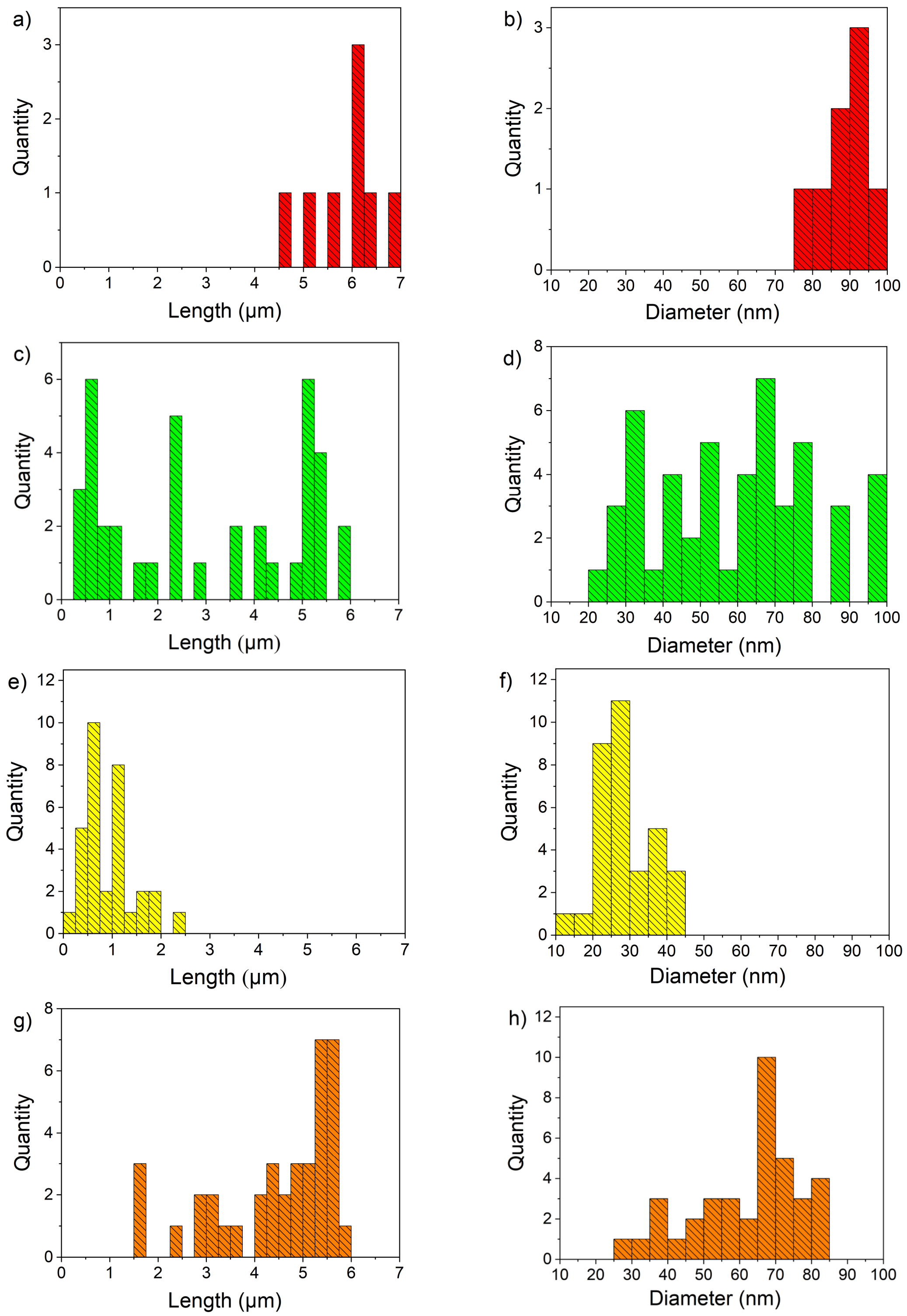

Figure S1. Histograms of NW length and diameters distribution for: (a, b) area I (0.052 pC/µm²); (c, d) area II (0.26 pC/µm²); (e, f) area III (10.4 pC/µm²); (g, h) unmodified area.
